# Exploring nurses' multitasking in clinical settings using a multimethod study

**DOI:** 10.1038/s41598-023-32350-9

**Published:** 2023-04-07

**Authors:** Yoojin Kim, Mi Ja Lee, Mona Choi, Eunhee Cho, Gi Wook Ryu

**Affiliations:** 1 National Evidence-Based Healthcare Collaborating Agency, Seoul, South Korea; 2 College of Nursing, Yonsei University, Seoul, South Korea; 3 Severance Hospital, Yonsei University Health System, Seoul, South Korea; 4 Mo-Im Kim Nursing Research Institute, College of Nursing, Yonsei University, Seoul, South Korea; 5 College of Nursing and Brain Korea 21 FOUR Project, Yonsei University, Seoul, South Korea; 6 Department of Nursing, Hansei University, 30 Hanse-Ro, Gunpo-Si, Gyeonggi-Do, 15852 South Korea

**Keywords:** Health services, Health care economics

## Abstract

Nurses often multitask in the process of managing patient care and communicating with healthcare providers simultaneously within a limited time, which can negatively affect patient care and safety. In this multimethod research, we conducted a time and motion study to record nursing activities using eye trackers for 23 participants (9 nurses and 14 patients). The frequency and duration of single and multitasking activities were analyzed. Additionally, we conducted focus group interviews (FGIs) with 12 nurses (2–5 nurses per group) to further investigate their multitasking experience. The total duration of the eye tracker recordings was 3,399 min. Daily nursing activities comprised 23.7%, 21.1%, and 12.5% of scheduled medication, documentation, and monitoring and measurement, respectively. Among these activities, nurses mostly carry out scheduled medication, monitoring, and measurement together. Three themes emerged in the FGIs: "Being involved in every little task regarding patient care," "Getting swamped by the complexity of symptoms and problems of the patients at a given time," and "Getting interrupted at work too often." Nurses performed multiple activities while cooperating with other healthcare providers and providing care to patients. It is important to create an environment where nurses can focus on essential nursing activities to improve patient safety.

## Introduction

Nurses perform multiple nursing activities simultaneously. They care for patients who often have a combination of acute and chronic symptoms, such as fever, dyspnea, high blood pressure, and cancer^1^. Furthermore, patients prefer to be cared for by registered nurses, who provide quality services and manage symptoms appropriately^1^. To meet patient needs and increase their satisfaction, nurses often handle multiple nursing activities at once, which is known as multitasking and is defined as performing two or more tasks simultaneously^2^. Multitasking may occur in nursing environments because of performing several activities within a short period.

Multitasking causes interruption to the workflow^3,4^, especially when there are various unexpected challenges interfering with nurses’ task, such as responding to patient questions or requests, lack of supplies, and equipment failure^5–7^. Multitasking is a risk factor for errors. While engaging in multiple tasks in a limited time, nurses can be distracted and lose focus on important aspects of their tasks, which may result in errors^7–9^. In most cases, nurses who are assigned to a task with a higher patient-to-nurse ratio experience increased multitasking owing to an increased demand for administrative duties, such as completing manual work and communicating with other health providers, in addition to the provision of direct care to a greater number of patients. Consequently, nurses may consider the time allocated to them insufficient to fully carry out the tasks assigned to them^10–13^.

Previous studies have approached this topic with different methodologies including observation, self-report, and interviews. Observation enables accurate data collection according to the type and duration of each task, however, it is labor-intensive and costly in addition to having variations in reliability across observers^14–16^. Moreover, having an observer on-site may influence workers’ behaviors and interfere with their duties^17^, which may lead to a biased result. The self-report methodology utilizes structured questionnaires to collect self-reported data, such as the type and duration of tasks. Although self-report enables the quick collection of a sizable amount of data, managing careless responses from participants and accurately understanding the context of nursing activities is difficult^13,18^.

The time and motion method is widely used in research^18^. It involves accurate observation of nurses’ work patterns, efficiency, and process of quick transitions between tasks^13^. Time and motion research using an eye tracker has been applied in various fields such as psychology, developmental psychology of infants and young children, cognitive linguistics, and medicine. In medicine, an eye tracker has been used to collect data regarding the behaviors of patients with dementia, Alzheimer's disease, and Parkinson's disease^18^. However, few studies have employed this method using an eye tracker in the nursing field.

In this study, we analyzed the contents and time required for multitasking using eye trackers and focus group interviews using multimethod research which combines qualitative and quantitative methods^19^. Analyzing only the work contents and required time could limit the understanding of the actual context of nurses' multitasking and their experiences. While few studies have been conducted on the contents and experiences of multitasking in the nursing environment, none of them existing studies explored focus group interviews. Therefore, focus group interviews are required to explain the context of the research results combined with video analysis from eye trackers of nurses' multitasking.

We confirmed the nurses' multitasking experience found in video analysis from eye trackers by further conducting focus group interviews to explore the context of those activities and the nurses' experience.

The aims of this multimethod study were (1) to identify the types and frequency of tasks and multitasking activities in nurses’ daily work and (2) to explore nurses’ experiences related to multitasking.

## Methods

### Study design

This study adopted a multimethod design. For the quantitative data, nurses’ activities were recorded using eye trackers and multitasking activities were analyzed thereafter. This part described the time and motion study. For the qualitative data, FGIs were conducted with nurses emphasizing the difficulties associated with multitasking based on the time and motion study. Data were collected from May to June 2019 at a tertiary hospital in Seoul, South Korea.

### Ethical statement

This study was approved by the Ethics Committee of the Severance Hospital Institutional Review Board (Y-2019-0002). All procedures were performed per relevant guidelines and regulations. Written informed consent was obtained from all participants.

### Study participants

For the time and motion study, convenience sampling was used to select participants at the internal medicine ward of a tertiary hospital in South Korea. We observed nurses’ activities in three shifts for 24 h: day, evening, and night. As there may be differences in the daily performance of nurses, observations were repeated for 3 to 4 days. In total, nine nurses participated in the study. The inclusion criteria were nurses working an 8-h shift and having a minimum of two years of nursing experience. In general, one nurse cares for 15 patients per shift in the internal medicine ward of tertiary hospitals in South Korea; thus, the targeted number of patient participants was 15 to 20.

To recruit nurses, our research team provided information on the study by attaching a poster on the ward’s bulletin board, and nurses who wished to participate were recruited. To recruit patients, nurse participants provided general information on the study to newly admitted patients during the study period. When a potential participant showed interest in the study, a member of the research team visited the ward and provided them with detailed information about the study. Patients who agreed to participate were assigned to the room where the nurse participant provided care.

For the FGIs, participants were purposefully sampled. The number of participants in this study was 12, which is within the recommended number of participants for FGIs^20^. The participants were nurses working shifts in the ward where the time and motion study was conducted. The inclusion criteria were nurses who worked eight-hour rotating shifts at the study ward with a minimum of two years of nursing experience. Researchers were informed about the nurses who met the inclusion criteria by the head nurse who approached nurses to identify those interested in the study. Subsequently, the study was explained, and informed consent was obtained from potential participants. The participants in the time and motion study were given the choice to participate in the FGIs.

### Outcome variables

In the time and motion study, outcome variables were nurses’ activities, which broadly included nursing activities and associated work. Based on the classification of standard nursing activities in South Korea, 15 major nursing activities, such as patient monitoring (e.g., checking vital signs), skincare, and timely medication, were used^21^. Based on the Basel Extent of Rationing of Nursing Care (BERNCA), we identified 23 related activities performed by nurses that did not directly affect patient outcomes, including patient transfer and retrieval of necessary equipment^21–23^.

### Data collection

In the time and motion study, nurses’ activities were recorded using the Tobii Pro Glasses 2 eye tracker (Tobii Technology), which has been proven safe to use in Europe (CE, Conformite Europeenne Mark) and Korea (Korea Standards Association)^24,25^. This eye tracker can store both video and audio data, and the audio data can clarify the situation by providing conversation recordings. In this study, eye trackers were used only to record nurses’ activities.

Nurse participants wore the eye tracker. A researcher who was not associated with the hospital conducting the study was in the ward to manage the eye tracker wearing, imaging device failures, and battery charging. The data were stored on an external hard drive with a secure device.

During the study, a sign reading “Recording using eye tracking equipment” was attached to the entrance of the station and the room where the recording was taking place. This was done to notify people that they might be filmed. Parts with recordings of unspecified persons were either deleted or blurred if they were difficult to delete.

FGIs were conducted at the participants’ desired time in the hospital’s conference room. The facilitator of the interviews was a researcher not affiliated with the hospital who had experience in qualitative research. Before the interview, the researcher explained the study and obtained written consent from the participants. Open-ended questions such as “How do you describe your experience of caring for patients when it comes to multitasking?” were used to encourage participants to talk about their experiences in a relaxed manner. During the interviews, an assistant took field notes and presented an overall summary of each interview before concluding it. Each interview lasted 60–90 min, after which the participants were given a small gift for their time spared for the interview. Once the interviews were finished, the facilitator and assistant discussed the data collected. The interview recordings were then transcribed verbatim. Data on the participants' general characteristics such as age, gender, and working experience were collected during the time and motion study and FGIs.

### Data analysis

For the quantitative study, the video data obtained with the eye trackers were analyzed using Tobii Pro Lab, considering shift start and end times, and the performed activities were tagged. Video analysis was performed by two nurses with master’s degrees and more than three years of clinical experience at a tertiary general hospital. To evaluate the validity of the video analysis, three out of nine shifts were analyzed preliminarily, and inter-observer concordance was evaluated based on the analysis of one randomly selected shift. Concordance was calculated as the percentage of recorded activities from the total number of activities. After confirming concordance, two researchers conducted three rounds of analysis to classify the nursing activities. In the first round, they recorded the nursing activities and their durations in seconds and classified the tagged contents according to the list of activities^21–23^. Nursing activities that were not observed during data analysis were excluded from the nursing activity category according to the BERNCA tool. Activities with an unclear classification or definition were marked, and each researcher’s analysis results were crosschecked to ensure their reliability and validity. Based on the final video analysis, both researchers independently classified multitasked activities, and disagreements in the classifications were resolved upon discussion with the remaining researchers.

Short nursing activities of less than 5 s were excluded from the analysis because of the difficulty to define them. Furthermore, the time taken for personal activities and travel time within or between wards were excluded from the analysis. The tasks performed after an activity, such as cleaning the thermometer after measuring the patient’s temperature, and cleaning up were not direct care-related activities, however, were considered as a continuum of the care provided. Thus, they were included in the activities.

To analyze the multitasking of nursing activities, two researchers independently classified the multitasked activities. The classification and analysis of consecutive nursing activities were discussed among the research team members. For example, some multitasked activities included discussing about a patient with another healthcare provider while entering data into the electronic medical record (EMR) and asking the patient about pain and discomfort while administering medication. Activities that could not be classified as a single activity, such as checking the amount of fluid and the injection site while measuring blood pressure with a cuff and measuring the pulse, were also considered multitasking. After classifying the tagged nursing activities using the classification tool, the frequency and duration of each activity were presented using descriptive statistics and were analyzed using the R software version 3.6.2.

To analyze nurses’ multitasking activities, the activities performed simultaneously were separated and their duration was calculated. First, as the nurses worked three shifts for 24 h, we observed each of the three shifts (day, evening, night) three times. Second, we summed the observed nursing activities for each shift group nine times (3 times during the day, 3 times during the evening, and 3 times during the night). Third, we divided the sum of all activities by three to calculate the nursing activity per day (based on 24 h).

FGI data were analyzed using the qualitative content analysis method proposed by Elo and Kangas^26^. After reading the transcripts and field notes several times, repeated and meaningful statements were underlined and contemplated. Meaningful units related to multitasking situations were categorized and clustered into themes that were more abstract. The themes and categories were listed separately and were rechecked by reading the transcripts to confirm that the major meaning units were included. The final themes and categories were extracted through a discussion between the researchers.

## Results

We first present the quantitative results, followed by qualitative results, as the latter help expand the results of the quantitative study. Nine nurses participated in the time and motion study, with three nurses each in day, evening, and night shifts, without dropout. All nurse participants were women. Their mean age was 30.44 years (SD = 5.1), and everyone had a bachelor's degree (n = 8, 88.9%) or higher (n = 1, 11.1%). The total work experience was 7.3 years (SD = 4.69). There were 14 patients assigned to the nurse participants and there were no dropouts.

The total duration of the video recording was 4,318 min. Of this, approximately 919 min (21.3%) were lost due to various reasons, such as when a recording paused after the participant took off the eye tracker, error in uploading the recording on the computer, and device malfunctioning. Finally, 3,399 min (78.7%) of video recording data were included in the analysis. In this, 1,481, 807, and 1,111 min of data were included for day, evening, and night shifts, respectively (Table 1).

**Table 1:** Video recording information.

	Day shift	Evening shift	Night shift	Total (%)
Recorded minutes	1,481.0	807.0	1,111.0	3,399.0 (78.7)
Lost minutes*	149.0	389.0	381.0	919.0 (21.3)
Total	1,630.0	1,196.0	1,492.0	4,318.0 (100.0)

*Lost when saving data on the computer or a failure to record because of device error.

### Reliability testing of the time and motion study

After excluding travel and personal time in the ward (180 min), the recorded data was 3, 219.4 min (53.7 h). Two researchers independently analyzed the video data. To evaluate the reliability of the analysis, approximately 1,578 min (26.3 h, 49.0%) of data for three out of nine shifts were analyzed preliminarily. Concordance was examined based on the analysis of 486 min (8.1 h, 15.1%) of data for a randomly selected shift. The degree of agreement between the analysts was confirmed using Cohen's kappa (0.97), which was found to be very high according to the criteria mentioned by Lanmdis and Koch ^27^.

### Analysis of nursing activities

Initially, 2,497 activities were identified for the analysis of nursing work. After excluding 433 short activities (22.1 min) lasting less than 5 s and 89 activities (107.7 min) involving personal time (e.g., eating, talking, resting), movement, and research, a total of 1,975 activities (3,219.4 min) were included in the analysis. Among these, 984 (49.8%) took place during the day shifts, 508 (25.7%) during the evening shifts, and 483 (24.5%) during the night shifts. Overall, 986 single activities (49.9%) consumed 2,234.7 min (69.4%). For multitasked activities, 989 (50.1%) were identified among which 849 (43.0%) involved two tasks, and 140 (7.1%) involved three or more tasks. Multitasking most frequently occurred during day shifts (2 tasks = 441 events, 44.8%; 3 or more tasks = 63 events, 6.4%), followed by evening shifts (2 tasks = 229 events, 45.1%; 3 or more tasks = 57 events, 11.2%) and night shifts (2 tasks = 179 events, 37.1%; 3 or more tasks = 20 events, 4.1%) (Table 2).

**Table 2:** Number and duration of nursing activities, per shift and 24-h period (N = 1,975).

Type	Total (%)		Single (%)		Multitasking (%)			
					2 tasks		3 or more tasks	
	n	Duration	n	Duration	n	Duration	n	Duration
Day	984 (100)	1,300.6 (100)	480 (48.8)	842.2 (64.8)	441 (44.8)	355.7 (27.3)	63 (6.4)	102.7 (7.9)
Evening	508 (100)	757.1 (100)	222 (43.7)	479.5 (63.4)	229 (45.1)	233.7 (30.9)	57 (11.2)	43.9 (5.8)
Night	483 (100)	1,161.9 (100)	284 (58.8)	913.0 (78.6)	179 (37.1)	207.8 (17.9)	20 (4.1)	41.1 (3.5)
Total	1,975 (100)	3,219.4 (100)	986 (49.9)	2,234.7 (69.4)	849 (43.0)	797.1 (24.8)	140 (7.1)	187.6 (5.8)

Duration is expressed in minutes.

For the 1,975 nursing activities, the frequency and time spent on each type were examined. Each activity within the multitasked nursing activities was included in the calculation of nursing activities. For multitasking, the total number of nursing activities was 3,104, which included 2,855 nursing and 249 associated activities. The time for each nursing activity involved in multitasking was calculated by dividing the multitasking time by the number of tasks. For example, if two tasks of medication administration and blood pressure measurement were performed for 30 s as multitasking, the time was divided between the two tasks, assigning 15 s to each task.

Regarding the duration, the top five nursing activities were documenting nursing care (22.9%, 738.1 min), scheduled medication (22.2%, 714.5 min), handover (15.3%, 491.9 min), monitoring and measurement (10.0%, 320.8 min), and housekeeping (9.0%, 289.6 min). The types of separate nursing activities performed includeddocumenting nursing care (24.8%, 554.7 min), handover (21.2%, 474.4 min), and scheduled medication (19.4%, 434.3 min). Moreover, approximately 65% of multitasked nursing activities were scheduled medication (28.5%, 280.2 min), documenting nursing care (18.6%, 183.4 min), and monitoring and measurement (17.9%, 176.6 min) (Table 3).

**Table 3:** Analysis of the frequency and time spent on nursing activities (single task and multitasking).

Categories*	Total				Single				Multitasking			
	n	%	Duration	%	N	%	Duration	%	n	%	Duration	%
Nursing activities	2,855	92.0	2,840.2	88.2	865	87.7	1,905.9	85.3	1,990	94.0	934.3	94.9
Monitoring and measurements	489	15.8	320.8	10.0	163	16.5	144.2	6.5	326	15.4	176.6	17.9
Skincare	7	0.2	5.4	0.2	–	–	–	–	7	0.3	5.4	0.5
Pain management	16	0.5	15.7	0.5	1	0.1	0.3	0.0	15	0.7	15.4	1.6
Comfort/talk with the patient	213	6.9	114.6	3.6	42	4.3	46.8	2.1	171	8.1	67.8	6.9
Teach/counsel patients and family	34	1.1	12.8	0.4	6	0.6	2.4	0.1	28	1.3	10.4	1.1
Treatments and procedure	107	3.4	129.3	4.0	36	3.7	86.9	3.9	71	3.4	42.4	4.3
Scheduled medication	802	25.8	714.5	22.2	263	26.7	434.3	19.4	539	25.4	280.2	28.5
Prepare patients and families for discharge	51	1.6	77.0	2.4	23	2.3	63.0	2.8	28	1.3	14.0	1.4
Documenting nursing care	506	16.3	738.1	22.9	148	15.0	554.7	24.8	358	16.9	183.4	18.6
Develop or update care plans	2	0.1	0.6	0.0	–	–	–	–	2	0.1	0.6	0.1
Coordinate patient care	502	16.2	183.6	5.7	117	11.9	70.8	3.2	385	18.2	112.8	11.5
Handover	103	3.3	491.9	15.3	59	6.0	474.4	21.2	44	2.1	17.5	1.8
Admission or transfer	2	0.1	0.7	0.0	–	–	–	–	2	0.1	0.7	0.1
Other (organizing and storing patients’ personal belongings, searching for patients, etc.)	21	0.7	35.3	1.1	7	0.7	28.1	1.3	14	0.7	7.1	0.7
Associated Work	249	8.0	379.2	11.8	121	12.3	328.8	14.7	128	6.0	50.3	5.1
Delivering and retrieving food trays	1	0.0	0.1	0.0	–	–	–	–	1	0.0	0.1	0.0
Ordering, coordinating, or performing ancillary services	1	0.0	0.5	0.0	–	–	–	–	1	0.0	0.5	0.1
Routine blood sampling	42	1.4	79.6	2.5	27	2.7	67.2	3.0	15	0.7	12.5	1.3
Transporting of patients	2	0.1	0.6	0.0	–	–	–	–	2	0.1	0.6	0.1
Housekeeping	177	5.7	289.6	9.0	88	8.9	259.1	11.6	89	4.2	30.6	3.1
Search for a supply or equipment	26	0.8	8.7	0.3	6	0.6	2.6	0.1	20	0.9	6.1	0.6
Total	3,104	100	3,219.4	100	986	100	2,234.7	100	2,118	100	984.7	100

Note: The frequency of multitasking nursing activities was calculated as separate nursing activity.

Multitasking nursing activity implies two or more nursing activities of 5 seconds or more.

Duration is expressed in minutes.

–, = not reported

The top five nursing activities in terms of time spent in a day were as follows: 23.7% (347.4 min) on scheduled medication, 21.1% (309.0 min) on documenting nursing care, 12.5% (183.6 min) on monitoring and measurement, 11.6% (169.8 min) on handover, and 7.4% (108.0 min) on housekeeping (Table 4).

**Table 4:** Duration of each nursing activity (Daily).

Categories	Minutes (%)		
	Total (%)	Single (%)	Multitasking (%)
Scheduled medication	347.4 (23.7)	144.6 (19.4)	202.8 (28.2)
Document nursing care	309.0 (21.1)	184.8 (24.8)	124.2 (17.3)
Monitoring and measurement	183.6 (12.5)	48.0 (6.4)	135.0 (18.8)
Handover	169.8 (11.6)	158.4 (21.3)	11.4 (1.6)
Housekeeping	108.0 (7.4)	86.4 (11.6)	21.6 (3.0)
Coordinate patient care	100.8 (6.9)	23.4 (3.1)	77.4 (10.8)
Comfort/talk with the patient	69.6 (4.8)	15.6 (2.1)	54.0 (7.5)
Treatments and procedure	61.2 (4.2)	28.8 (3.9)	32.4 (4.5)
Routine phlebotomy	33.6 (2.3)	22.2 (3.0)	11.4 (1.6)
Prepare patients and families for discharge	31.2 (2.1)	21.0 (2.8)	10.2 (1.4)
Other (organizing and storing patients’ personal belongings, searching for patients, etc.)	14.4 (1.0)	9.6 (1.3)	4.8 (0.7)
Pain management	13.8 (0.9)	0 (0.0)	13.2 (1.8)
Teach/counsel patients and family	10.2 (0.7)	0.6 (0.1)	9.0 (1.3)
Looking for supplies or equipment	4.8 (0.3)	0.6 (0.1)	4.2 (0.6)
Skincare	4.8 (0.3)	0 (0.0)	4.8 (0.7)
Transporting of patients	0.6 (0.0)	0 (0.0)	0.6 (0.1)
Prepare patients and families for admission or transmission	0.6 (0.0)	0 (0.0)	0.6 (0.1)
Develop or update care plans	0.6 (0.0)	0 (0.0)	0.6 (0.1)
Arranging discharge referrals and transportation	0.6 (0.0)	0 (0.0)	0.6 (0.1)
Delivering and retrieving food trays	0	0	0
Total	1,464.0 (100)	745.2 (100)	718.8 (100)

### Multitasking

The types and durations of multitasked activities were analyzed, with the duration of each activity calculated by dividing the total duration by the number of activities involved in that multitasked activity at a given time. The total duration of multitasking was 984.7 min. Scheduled medication as well as monitoring and measurement were the most frequent activities multitasked together, with approximately 302.3 min (22.2%) spent on these activities. The participants spent 165.8 min (12.2%) on scheduled medication activities while documenting nursing care, and 135.6 min (10.0%) were spent on documenting nursing care while coordinating patient care. Approximately 99.9 min (7.4%) were spent on scheduled medication activities and 90.2 min (6.6%) were spent on monitoring and measurement of patient surveillance, while comforting/talking with the patient. The nurses spent 563.8 min (41.5%) on other activities while performing scheduled medication activities, including monitoring vital signs and oxygen saturation, informing patients about the medication and testing schedule for the day, checking patients’ symptoms, documenting nursing care, and continuously communicating patient information with other healthcare providers.

### Focus group interviews

Four FGIs were conducted with 12 nurses. Each group consisted of two to five nurses. Five of the nurses involved in FGIs participated in the time and motion study as well. All participants were women, with a mean age of 32.17 years (SD = 9.13). Their mean clinical experience was 9.1 years (SD = 4.9), with a range of 2–22 years. The mean years of experience at the current ward was 6.3 years (SD = 3.2). Regarding education level, ten participants had a bachelor’s degree, one had a master’s degree, and one had an associate degree. Multitasking-related challenges and nurses’ experiences when working rotating shifts in the tertiary hospital were analyzed using a qualitative data analysis method, and nine categories under three themes were identified.

The three themes were “Being involved in every little task regarding patient care,” “Getting swamped by the complexity of symptoms and problems of the patients at a given time,” and “Getting interrupted at work too often.” In the first theme, nurses mentioned that all communication among healthcare providers, staff, and patients passed through them. This required them to multitask and pause whatever they were doing at the time. In addition to nursing activities, nurses had to address patients’ requests regarding getting documents for them as well as other tasks such as changing bed sheets and organizing the fluids stock room. They mentioned that patients, staff, and nurses had different ideas regarding the scope of nurses’ work, and the ambiguous work description further increased their workload.

Regarding the second theme, “Getting swamped by the complexity of symptoms and problems of the patients at a given time,” nurses mentioned challenges regarding patients’ comorbidities when multitasking. Patients admitted to a tertiary hospital generally have various comorbidities, and those with similar diagnoses are often given different medications and tests. They mentioned challenges regarding rapid changes in patients’ conditions and dealing with the subsequent changes in prescriptions and care. It also challenged nurses as they had to quickly care for patients within limited working hours.

Concerning the theme “Getting interrupted at work too often,” nurses mentioned that their workflow was interrupted by phone calls, patients, caregivers, and doctors’ rounds (Table 5).

**Table 5:** Themes and categories of multitasking-related difficulties of nurses.

Themes	Categories
Being involved in every little task regarding patient care	Being a bridge in communication between other healthcare providers and patients
	Going beyond the role of nursing
Getting swamped by the complexity of symptoms and problems of the patients at a given time	Identifying, understanding, and managing complex patient symptoms quickly
	Having to promptly manage rapidly changing symptoms
	Delayed work because of frequent changes in the doctor’s order
	Stacking up on work while caring for delirious patients
Getting interrupted at work too often	Phone keeps ringing
	Impatience of patients and caregivers
	Having to stop work and participate in doctors' rounds

## Discussion

In the present study, FGIs were conducted to gain an in-depth understanding of the context of a time and motion study. Through the FGI, the difficulties and context related to multitasking and nursing activities were explored. The percentages of total time spent performing nursing activities per shift were 49.9%, 25.7%, and 24.4% in day, evening, and night shifts, respectively, which were similar to the rates reported in a previous study (day: 36.7%, evening: 31.1%, and night: 32.2%)^22^. Considering the nursing work environment, most of the patient-related work was conducted during the day, and the duration of nursing activities in the day shift was the longest.

Previous studies obtained different results regarding the most frequent nursing activities. This study showed that the percentages of the duration of nursing activities were scheduled medication (23.7%), documenting nursing care (21.2%), monitoring and measurements in patient surveillance (12.5%), and handover (12.5%). In a previous study, nursing activities were reported as follows: 24.6% for record keeping, 24.0% for handover, 13.9% for medication administration, 8.9% for measurements (e.g., vital signs), and 5.3% for communication^28^. In this study, scheduled medication included the process of preparing and collecting medications from the pharmacy, reclassifying drugs, and recording the time on the administration card for each patient before administering medication. Thus, the variations may have been influenced by differences in the definition and classification of medication-related nursing activities. The findings also suggest that medication administration is an important activity that accounts for a substantial portion of nursing activities.

Scheduled medication as well as monitoring and measurements were most frequently multitasked together and accounted for 302.3 min (22.2%), suggesting that nurses were assessing and monitoring patients’ vital signs while administering medications. This result indicated that these two activities were closely associated and that nurses considered various situations and factors when performing activities. For example, nurses checked the dose and rate of fluid being infused and asked the patient whether they faced any discomfort while measuring their blood pressure using a cuff. Furthermore, nurses talked about the oral medications that the patient would subsequently take while measuring their vital signs, such as blood pressure and body temperature, after which they administered the oral medications. In this respect, the theme “Getting swamped by the complexity of symptoms and problems of the patients at a given time,” and the category “Delayed work because of frequent changes in the doctor’s order” emerged in the FGIs. Nurses’ work was delayed because of changes in the medication according to changes in the patient’s symptoms, which led to multitasking.

Therefore, nurses conducted medication and monitoring activities while checking patient symptoms. Severe adverse drug reactions can lead to prolonged hospitalization, complications, and death, thereby threatening patient safety^1^. The 2018 Patient Safety Statistical Yearbook reports that 28.1% of patient safety accidents are caused by dosing errors, doubling from 1,075 cases in 2017 to 2,602 cases in 2018. Scheduled medication has an important impact on symptom management and patient safety^28,29^. Therefore, nurses spend a lot of time administering medications and monitoring patients to reduce medication errors. Our findings also showed that nurses’ work is not simple, and although nursing activities can be classified according to the categories of vital sign measurement, scheduled medication, and providing explanations to patients, they vary and cannot be measured independently.

Scheduled medication activities and documenting patient records were also frequently performed together accounting for 165.8 min (12.2%), showing that certain activities, including using the EMR to confirm the prescription and medication, and signing it off while recording notable events or patient’s reaction to the administered medication, were linked to each other. In a previous study, nurses multitasked during medication administration, there were approximately two interruptions per hour, and 27% of interruptions occurred during scheduled medication activities^11,30^. In this study, 3,219.4 min of nursing activities were performed over three days. In multitasking, two and three or more tasks were performed for 24.8% and 5.8% of the time, respectively. Considering that nurses multitasked for approximately 4 h out of the 8.1 h (486 min) spent on nursing activities, the total time spent on nursing activities accounted for approximately 12 of 24 h. One of the themes that emerged from the FGIs was “Getting swamped by the complexity of symptoms and problems of the patients at a given time,” and participants mentioned that their work involved arranging different tests, providing pre-test care, and dealing with changes in drug prescriptions because of patients’ complex comorbidities. Therefore, it can be inferred that complex symptom management and multitasking were challenging for nurses.

In terms of associated work, nurses spent 14.7% of their time on single activities and 5.1% on multitasking, which included housekeeping and routine blood sampling. As revealed in the FGIs, the category “Going beyond the role of nursing,” indicated that they were required to address patients’ or caregivers’ complaints immediately despite this being beyond their scope of work. Associated work causes nurses to develop a negative image of their role identity and may influence burnout and job dissatisfaction^11^. Therefore, it is necessary to transform the nursing work environment so that nurses can focus on nursing activities with less associated work.

“Coordinate patient care” was performed 502 times (16.2%), which took 173.6 min (5.7%). One of the categories identified in the FGIs was “Having to bridge all communication among healthcare providers, staff, and patients.” These results showed that interdisciplinary communication was important for sharing information and making disease-related decisions. As for sharing information and making disease-related decisions, multitasking (e.g., having to repeatedly confirm the information because of inadequate interdisciplinary communication and/or exchanging information between care teams) may interrupt nurses’ work and lead to an increased workload.

Observation and self-report have been mainly used to analyze nurse workloads. With observation, it is difficult to generalize the results because of differences in observers' reliability^31,32^. Self-reports are convenient but may be characterized by insincerity and a lack of understanding of activities by nurses^33^. To improve the objectivity of data collection, audio and video recording technology is used to supplement the observation method^34^.

It is also necessary to reduce nurses' multitasking in drug preparation and administration so that they can focus on their specific tasks. Similarly, hospital management should improve patient safety by developing technologies and information systems that can prevent medication errors. For example, nurses can use real-time wireless technology to scan barcodes, use automated dispensing cabinets and pump channels, and employ systems that allow real-time, non-bedside monitoring^35,36^. In addition, when planning a nurse staffing the number of nurses should be calculated with respect to multitasking.

This study revealed that nurses spent 11.8% of their total work time engaging in associated work, such as delivering and retrieving food trays, performing housekeeping, and searching for a supply or equipment. Hospital and healthcare organizations should develop strategies that will allow nurses to primarily focus on patient care. For example, tasks that are not directly for nurses could be assigned to unit clerks.

This study was the first to examine multitasking among nurses based on video data collected using eye trackers. The findings were more reliable because different researchers identified the types and durations of activities and resolved discrepancies without relying on a sole observer's judgment. Additionally, it was possible to confirm the various nursing activities performed through multitasking, which could not be done in previous studies on nursing activity. Moreover, the context of nurses’ multitasking experience was explored through FGIs.

This study has a few limitations. First, the study was conducted in a single ward in one hospital. Therefore, it is difficult to generalize the findings to other medical disciplines such as surgery, pediatrics, intensive care unit, and emergency department. Second, in the time and motion study, there might have been a Hawthorne effect, which refers to acting differently when knowing that one is participating in a study^37^. In this study, while nursing activities were being recorded, participants might have improved their performance or increased their productivity. Due to the continuity and interrelation of nursing activities, it was difficult to distinguish the beginning and end of each nursing activity. Further studies are needed to standardize the multitasking analysis methodology.

Future research on nursing activities should be conducted through advanced research methods such as eye trackers. In addition, further research on multitasking should be conducted in different types of wards and hospitals.

## Conclusions

In this multimethod research, we conducted a time and motion method using eye trackers and FGIs to gather data and demonstrate the phenomenon of nurses’ multitasking. We found that the eye tracker was effective in measuring the objective activities of healthcare providers, and this method may be useful in future research.

From the results of this study, we obtained objective evidence that nurses' activities are consequential and interrelated. Nurses perform various nursing activities in multitasking, such as providing scheduled medication, monitoring, and communicating. As a considerable number of nurses' multitasking activities occur during medication administration and monitoring related to complex symptoms, it is imperative to understand patient safety from the perspective of multitasking. Moreover, nurses are often interrupted in their work while dealing with the complexity of patients' symptoms and interacting with caregivers and other healthcare providers.

Developing and using technologies, information systems, and non-bedside monitoring can reduce multitasking and improve patient safety. Moreover, by using support systems such as unit clerks, associated works or unnecessary related tasks would reduce, allowing nurses to focus mainly on patient care. Hospital management should adjust nurses’ work policy to reflect the appropriate number of patients per nurse to improve the quality of care and the nursing work environment.

The findings of this study with relevant suggestions would help improve the quality of nursing care and patient safety.

## Supplementary Information

Supplementary Information.

## Data Availability

The datasets generated and analyzed during the current study are not publicly available because of privacy or ethical restrictions. However, they are available from the corresponding author upon reasonable request.
