# Supplementary material for: Exploring nurses' multitasking in clinical settings using a multimethod study
Source: Sci Rep. 2023 Apr 7;13:5704. doi: 10.1038/s41598-023-32350-9 (PMC10082008; doi:10.1038/s41598-023-32350-9)
Supplement: Supplementary file 1 — Supplementary Information. [file 41598_2023_32350_MOESM1_ESM.docx]

Supplementary Table S1. Nursing activities and associated work definition

| **Category^*^** | **Definition** |
| --- | --- |
| **Nursing activities** | |
| Monitors and measurements | Take vital signs, I/O check (e.g., JP/PCD,PCD), check consciousness, measure oxygen saturation, measure body weight and height, blood glucose test  Includes emptying bag to measure I/O and discarding urinary drainage bag |
| Skin care | Includes wound care and dressing performed by a nurse |
| Oral hygiene | Oral care performed by a nurse |
| Pain management | Classified into pain assessment (monitoring) and administration of analgesics, but pain assessment and medication administration were classified into a single pain management category |
| Comfort/talk with patient | Includes all conversations with the patient or caregiver |
| Teach/counsel patients and family | Explaining preoperative and postoperative symptom management, tests, and procedures |
| Treatments and procedure | 1) Care (insertion and management of central venous catheter, peripheral venous catheters), tube insertion and management, Drainage (replacing CTD bottle, checking patency), surgery and procedure-related care, ice or hot pack, CSF tapping, Bone marrow Bx, paracentesis assist (pleural effusion fluid, ascites)  2) Special care: CPR, isolation care (isolation /reverse isolation), physical restraints, fall prevention, postmortem care |
| Scheduled medication | 1) fluid IV, TPN infusion and management, IV medication administration, oral medication administration and checking administration, administer narcotic analgesics, anticancer agents, calculate anticancer agent dosage, intramuscular, intradermal, subcutaneous injection, blood transfusion  2) Includes checking 5 Right for medication and preparing for medication administration: check water, print medication barcode, manage medication card, manage fluid lines |
| Prepare patient and families for discharge | Includes checking and printing EMR and giving medication instructions for patients being discharged |
| Documenting nursing care | 1) Check and search physician’s prescription: electronic documentation of regular and additional prescriptions  2) Manage medical records (e.g., print, prepare,)  Checking EMR prescription, signing off a medication, or check for additional prescriptions while checking the drug is considered to have been performed with documentation activity |
| Develop or update care plans | Added nursing intervention due to newly identified problem (Isolation or counter-quarantine nursing intervention is performed after infection is confirmed) |
| Coordinate patient care | Exchange opinions with physician (including phone calls, text messages)  Coordinate and refer to a different department (communication with other nursing units, medical engineering, facility, IT department, nurses’ aide, assistants, and transporting staff)  Exchange opinions among nursing staff (consult and discuss with nursing staff regarding a patient)  Answer and direct phone calls (direct phone call to someone else, answer misdirected phone calls), participate in rounds |
| Handover | Unit handover, individual handover, check ward bulletin |
| Admission or transfer | Includes preparation, handover, and cleaning up after patient when patient is transferred into or out of ward |
| **Associated Work** | |
| Delivering and retrieving food trays | Delivery and retrieval of patient meals |
| Ordering, coordinating, or performing ancillary services | Transfer referral and management  Includes caregiver education for transfer, preparing documents for transfer, checking and preparing equipment, and transporting healthcare staff needed for transfer |
| Routine blood sampling | Routine and additional blood sampling |
| Transporting of patients | Includes helping patient up or transporting patient for a test |
| Housekeeping | 1) Check supplies (check inventory, purchase, and receive supplies/equipment/devices)  2) Organize and manage ward supplies (prepare supplies for care, supply handover and inspection)  3) Maintain cleanliness of the nursing unit  4) Manage equipment and wash equipment (change reagents and record papers for test equipment)  5) Includes patient’s request for exchanging linen or additional linen  6) Manage drugs and drug requests at the ward  7) Assign patient rooms |
| Search for a supply or equipment | Nurses are required to perform work on behalf of nurses after regular office hours of another department, such as picking up medicines after hours from a pharmacy |
| Search for a supply or equipment (e.g., pillow, dressing, iv holder) | Includes borrowing supply from another department when it runs out |
| Other | 1) Organizing and storing patient’s personal belongings  2) Searching for patients |
| Bx, biopsy; CPR, cardio-pulmonary resuscitation; CSF, cerebrospinal fluid; CTD, chest tube drainage; EMR, electronic medical record; I/O, intake output; IT, information technology; IV, intra-Venous; JP, Jackson-Pratt drain; PCD, percutaneous catheter drainage; TPN, total parenteral nutrition  * This category was classified according to standard nursing activities in Korea (Park, 2000) and the Basel Extent of Rationing of nursing Care (BERNCA) instrument (Cho et al., 2016; Schubert et al., 2007). | |
